# Low-level light therapy reduces platelet destruction during extracorporeal circulation

**DOI:** 10.1038/s41598-018-35311-9

**Published:** 2018-11-16

**Authors:** Anna Drohomirecka, Alicja Iwaszko, Tomasz Walski, Aleksandra Pliszczak-Król, Grzegorz Wąż, Stanisław Graczyk, Katarzyna Gałecka, Albert Czerski, Jolanta Bujok, Małgorzata Komorowska

**Affiliations:** 1 Department of Heart Failure and Transplantology, Institute of Cardiology, Warsaw, Poland; 2 Regional Specialist Hospital in Wrocław, Research and Development Centre, Wrocław, Poland; 3 Department of Immunology, Pathophysiology and Veterinary Prevention, Wrocław University of Environmental and Life Sciences, Wrocław, Poland; 4 Department of Biomedical Engineering, Wrocław University of Science and Technology, Wrocław, Poland; 5 “Medinet” Lower Silesian Centre for Heart Diseases, Wrocław, Poland; 6 Department of Animal Physiology and Biostructure, Faculty of Veterinary Medicine, Wrocław University of Environmental and Life Sciences, Wrocław, Poland

## Abstract

Extracorporeal circulation causes many deleterious effects on blood cells. Low-level light therapy (LLLT) in the red/near-infrared spectral range is known for its cytoprotective properties but its use during cardiopulmonary bypass (CPB) has not yet been studied. We aimed to assess whether LLLT protects platelets during CPB. 24 pigs were connected to 1-hour-CPB and observed for the next 23 hours. In 12 animals, blood circulating through the oxygenator was treated with LLLT. Platelet count and function were monitored throughout the experiment. The decrease in platelet count was greater in the control group, especially during CPB and after 24 hours. In LLLT group CD62P expression remained quite stable up to the 12^th^ hour of the experiment, whereas in the control group it continuously decreased till the end of observation. Platelets in the control group were more prone to aggregation in the postoperative period than at the beginning of the experiment, whereas platelets in the LLLT group aggregated similarly or less intense. Limitation of platelet loss, pattern of aggregation and CD62P expression suggest that LLLT may stabilize platelet function during CPB and diminish the negative effects associated with the interaction of cells with an artificial surface.

## Introduction

The heart-lung machine is a critical device in modern cardiac surgery. Up to 0.5 million cardiac operations using extracorporeal circulation (ECC) are performed annually in the United States^1^. Cardiopulmonary bypass (CBP) replaces the function of the patient’s heart and lungs for the duration of surgery, making hours-long and complicated heart operations possible. Unfortunately, contact of the blood with an artificial surface leads to many adverse pathophysiological processes, i.a.hemostasis disorders. During extracorporeal circulation, activation and adhesion of both platelets (PLT) and leukocytes occur, which consequently leads to leukocytes and PLT aggregation and thrombus formation^2^. On the other hand, non-physiological shear stress is thought to induce shedding of the receptors glycoprotein (GP) Ibα, and GP along with fragmentation of von Willebrand factor (vWF) which may increase the risk of bleeding^3,4^. Therefore, stable PLT function is crucial for maintenance of hemostasis.

In this study, we aimed to evaluate whether red/near-infrared (R/NIR) low-level light therapy (LLLT) impacts PLT activation during and after ECC in a swine model of CPB.

It was previously shown that near-infrared radiation reduced osmotic fragility of erythrocytes^5^. Moreover, Itoh *et al*.^6^ demonstrated photochemical protective effects when using LLLT in a model of *ex vivo* extracorporeal circulation - RBCs were exposed to a He-Ne laser radiation for four hours and a decrease in intracellular ATP-depletion, erythrocyte deformability loss, and hemolysis was seen. Other studies^7,8^ have reported that R/NIR radiation increases the electrochemical potential of erythrocytes, which may directly contribute to the decrease of their aggregation potential during rouleaux formation^9^. In addition, modulation of membrane enzyme activity has been repeatedly demonstrated^10,11^. Moreover, RBC membrane lipid peroxidation in response to ozonation was reduced in the presence of NIR irradiation^8^. If by analogy, LLLT reduced the fragility of PLT to stimuli generated during CPB and consequently stabilized their activity, LLLT therapy could be used to attenuate PLT-related coagulation disorders.

## Material and Methods

### Experimental system/Experimental design

The study was carried out on 24 young adult female pigs (aged 5 months, Polish Landrace, average weight 94.3 ± 3.2 kg). Animals originated from a single farm (The National Research Institute of Animal Production, Experimental Station in Pawłowice, Poland), were clinically healthy and, apart from vaccination against rosacea (Suibiovac Ery, Biowet Drwalew, Poland), did not receive any drugs before the experiment. The experiment consisted of one-hour venous-arterial ECC from cervical access using a heart-lung machine without any additional surgical procedures. Animals were divided into two experimental groups of 12 individuals: the control group and the LLLT group, in which the blood flowing through the oxygenator was exposed to R/NIR light during the entire ECC period. Platelet function and activation was evaluated at multiple time points during and up to 23 hours after ECC (see paragraph “Collection of blood samples and PLT preparation”) and compared between the control group and the LLLT group. Platelet function was characterized by population size (cell count), mean platelet volume and level of antagonized aggregation by adenosine diphosphate or collagen. Platelet activation was measured by level of CD62P expression, an activation-dependent surface receptor. After 24 h from the start of ECC, all animals were euthanized by rapid injection of 60 mg/kg Pentobarbital (dose accordant with producer guidelines, Biowet Puławy, Puławy, Poland) through a central catheter.

### The procedure of extracorporeal circulation (ECC)

Premedication was achieved with intramuscular injection of ketamine (10 mg/kg, Bioketan, Vetoquinol Biowet Puławy, Poland), dexmedetomidine (10 μg/kg, Dexdor, Orion, Espoo, Finnland) and diazepam (20 mg/100 kg, Relanium, Polpharma SA, Poland). After obtaining peripheral venous access further drug administration was performed intravenously (*iv*). Anesthesia was induced and maintained with propofol (0.2 mg/kg/min, *iv*, Scanofol, ScanVet, Poland), fentanyl (50 μg/kg/10 min, *iv*, SA Polfa, Poland), ketamine (10 mg/kg/30 min, *iv*), diazepam (0.2 mg/kg/30 min, *iv*). To provide airway patency the animals were intubated and connected to a respirator (Wowo 500 Veterinary Anesthesia Machine, China). Respiratory parameters (CO_2_ partial pressure, O_2_ partial pressure, saturation and respiratory rate) were monitored. The animals were mostly self-breathing, however, mechanical ventilation was used if respiratory arrest lasted longer than a minute or elevation of CO_2_ concentrations were observed in the exhaust phase above 60 mmHg. General anesthesia time (including the preparation of vascular accesses, connection and performing extracorporeal circulation, decannulation, and blood collection after the procedure) was approximately two and a half hours.

Extracorporeal blood flow was carried out at normothermia using a Stoeckert 41-40-50 heart-lung machine (Sorin Group, Milan, Italy) with roller pumps. The study used disposable perfusion sets Maquet VKMO 78000 (Maquet, Rastatt, Germany), consisting of tubing made from polyurethane and silicone, cardiotomic reservoirs, VHK 2000, with a volume of 4200 mL made of polycarbonate membrane oxygenator Quadrox-i Adult and aortic filter. The oxygenator was connected by means of rigid drains with a Dual Heater Cooler HCU-20 water pump (Maquet, Rastatt, Germany). Cannulas for ECC were introduced through the neck vessels (jugular vein and carotid artery at the same side) after being surgically exposed. Local infiltration anesthesia of skin and subcutaneous tissue was achieved with the use of 2% procaine hydrochloride with 0.005% adrenaline (Biowet Drwalew SA, Drwalew, Poland). The perfusion set was pre-filled with a crystalline solution (priming), which included 1000 mL of Ringer’s fluid (Baxter Polska, Warsaw, Poland) and 500 mL of polyelectrolyte fluid (Baxter Polska, Warsaw, Poland), followed by addition of 3000 IU unfractionated heparin (heparinum WZF®, Polfa Warszawa, Poland). Additional doses of heparin were administrated when the activated clotting time (ACT) (ACT Analyzer Actalyke Mini II & MAX-ACT tubes, Helena Laboratories, Beaumont, USA) did not exceed 200 seconds before the start of each CPB, to prevent blood clotting in the extracorporeal circuit. During CPB, the main pump expenditure was maintained at a level of 40 to 50% of the cardiac output calculated individually for each animal. The air/oxygen mixer setting was 55%, with an oxygen flow of 2 to 3 L/min, which was enough to achieve normal perfusion pressure and blood saturation. ECC lasted one hour in each case. The weaning from CPB consisted in clamping of the venous drain and gradual filling of the arterial vessel bed with oxygenated blood under the control of arterial blood pressure. After CPB 0.8 mg of protamine sulfate (Prosulf, Wockhardt, UK; 10 mg/mL) for every 100 units of previously administered heparin (prime heparin not included) was given to achieve normalization of the ACT. The vascular cannulas were then removed and the vessels were closed with a synthetic suture, an absorbable form of 4.0 polydioxanone (PDS II, Ethicon LTD, Livingston, UK). The subcutaneous layer and skin were then sutured.

### Extracorporeal blood low-level light treatment

Extracorporeal blood irradiation was performed as was described previously^12^. We choose the oxygenator as a element of CPB optimal for irradiation. The hollow-fiber oxygenator is characterized by a large blood-to-surface area so it is a major site of the PLT activation. Furthermore, its rectangular cuboid shape determined the opposite, coaxial position of two R/NIR emitters in relation to the oxygenator, so that it irradiated both transparent to R/NIR walls made of polycarbonate (PC) which are also transparent in the visible spectrum range, as their primary function is to enable a visual assessment of the risk of excessive clot formation on the gas or heat exchange membranes. Each R/NIR emitter included nine low-voltage halogen burners with IRR coating in glass reflector and cover disks (MR11 + C GU4 30°, 20W, 12V, ANS Lighting, Warsaw, Poland), optical band-pass filter IFG 098 (Schneider-Kreuznach, Bad Kreuznach, Germany) and optical diffuser placed in a housing made of acrylonitrile butadiene styrene (ABS). Heat from the emitter was discharged by means of two fans (FD2510D12MB, F&F, Zhejiang, China). The adjustment of irradiance was carried out using an autotransformer (PowerLab VT5-1, Guangdong, China).

**Figure 1:**
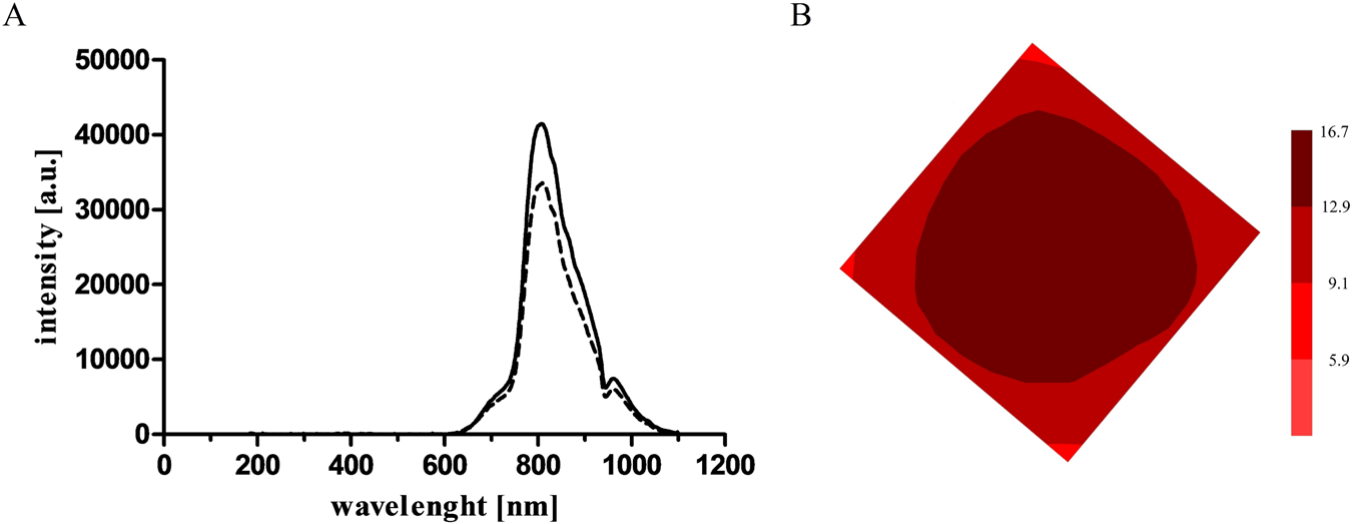
(**A**) The emission spectrum of the R/NIR irradiator on the surface (solid line) and after passing through the transparent quadrilateral wall of the oxygenator housing (dashed line). (**B**) The irradiance distribution measured under the exposed surface of the oxygenator housing wall expressed in mW/cm^2^.

The irradiation of blood flowing through the oxygenator was performed with non-coherent, unpolarized R/NIR electromagnetic waves in the range of 750 ÷ 1100 nm with a maximum intensity of 800 nm as shown in Fig. 1A. Fiber optic spectrophotometric system (Avaspec-3648 spectrophotometer, fiber optic FC-UV600-2, lens VIS-NIR 0.25NA SMA H2, software AvaSoft 7.3, Avalight BV, Apeldoorn, The Netherlands) was used to record spectra produced by R/NIR emitter. The irradiance distribution under the exposed surface of the oxygenator housing wall was measured with the radiometer (OPTEL, Opole, Poland) (Fig. 1B). The mean irradiance amounted to 12.8 mW/cm^2^. The dose of R/NIR radiation was adjusted individually depending on an animal body weight to obtain an absorbed dose of 1 J/cm^3^. Dose selection was based on the analysis of previously published data, including results of *in vitro* studies on the assessment of R/NIR radiation of different spectral ranges, irradiance levels and exposure time of blood cells^6–18^. It was shown that a dose from 0.044 to 1.45 J/cm^3^ inhibits platelet activation. Simultaneously, the radiation reduced erythrocytes destruction (hemolysis) in the tested samples^14^ Application of the dose below the indicated lower limit was ineffective, while the upper limit was the highest tested dose, and showed no signs of cytotoxicity. Thereby these results, to a large extent, are in line with the concept of a biphasic dose response in photobiomodulation^15^, however further studies should be carried out to define the upper dose threshold until a negative response is finally achieved.

### Collection of blood samples and PLT preparation

To minimize the animal stress all samples were taken through a central venous catheter which was inserted during general anesthesia at the beginning of the experiment and was maintained throughout the experiment. Blood samples were drawn at six defined time points: before the start of ECC (T0), 30 min after initiation of ECC (T0.5), directly after discontinuing ECC and ACT normalization (T1), and further 6 (T6), 12 (T12), and 24 (T24) hours after the start of ECC.

Depending on the type of assay, blood was collected in a test tube containing 3.8% sodium citrate (F.L. Medical SRL, Torreglia, Italy) in a ratio of 1:9 (anticoagulant: blood) or K_2_EDTA (Profilab s.c., Warsaw, Poland). The PLT count and their volume were measured in the whole blood samples using a hematologic analyzer (PE-6800 vet, Procan Electronics INC, China).

Expression of CD62P (P-selectin) receptors was evaluated on isolated PLT. Within one hour after collection, citrated pig whole blood was centrifuged at 150 × g for 10 min at room temperature to obtain PLT-rich plasma (PRP). The PLT-rich plasma supernatant was gently removed with a plastic pipette and transferred to another plastic tube. The PLTs were isolated by further centrifugation at 1200 × g for 10 min at room temperature and washed twice in RPMI-1640 (Sigma Aldrich, St. Louis, USA). For cytometric studies, samples of the concentrated PLT, 10 × 10^6^/mL, were used. To activate the PLT, thrombin was added to the test tube (Bio-Ksel System TT, Bio-Ksel, Grudziadz, Poland), and the PLT were incubated for 3 min at 37 °C. The thrombin concentration was 1 U/mL which is required for maximal porcine PLT activation. After the incubation, the PLT were washed twice using RPMI-1640.

### Analysis of CD62P (P-selectin) expression

100 µL of a suspension containing 1 × 10^6^ activated PLT was incubated for 30 min at room temperature in the dark with 10 µL of monoclonal, fluorescein-conjugated mouse anti-human CD62E/CD62P antibody (MCA 883 F; AbD Serotec, Oxford, UK). The cross-reaction with the porcine platelet CD62P was previously demonstrated in our pilot-studies. A monoclonal fluorescein (FITC)-labeled mouse IgG1 antibody was used as the negative control (MCA 928 F; AbD Serotec, Oxford, UK). Thereafter, PLT were washed twice by addition of 2 mL of RPMI-1640. After centrifugation at 1200 × g for 5 min, the PLT were resuspended in 500 µL of RPMI-1640 in preparation for flow cytometry.

### Flow cytometry analysis

Fluorescence was analyzed within 30 min using the FACS Calibur (Becton Dickinson Co, Rutherford, USA) and WinMDI 2.9 software (J. Trotter, The Scripps Research Institute, La Jolla, CA, USA). The PLT population was identified and gated according to their characteristic forward scatter (FSC) and side scatter (SSC) properties. The mean fluorescence intensity (MFI) was analyzed on a 4-decade log scale (1–10000); 10,000 cells were analyzed for each sample.

### PLT aggregation test

Impedance whole blood aggregometry was estimated using dual-channel lumi-aggregometer (model 700, Chrono-log, USA). A sample of 450 μL of blood and 450 μL of PBS was incubated for 5 min at 37 °C, followed by induction of aggregation by adding the agonist adenosine diphosphate (ADP; at 10 µM (#384, Chrono-log, USA) and 50 µl of 3 mM CaCl_2_ (POCH, Poland)) or collagen (2 µg/ml (#385, Chrono-log, USA)). Impedance changes were monitored for 6 min after addition of the agonist. The area under the curve (AUC) was analyzed at the individual measurement points versus T0.

### Statistical analysis

Bootstrap analysis of variance was performed, as described previously, to evaluate whether changes in the values of individual parameters during ECC were significant^16,19^. Briefly, detection of the changes in a time-series model was performed by generation of 2000 bootstrap samples using random sampling with replacement from the original dataset for each measured parameter examined separately for each group. Then, the mean for each bootstrap sample was calculated and the sampling distribution of means using bootstrap estimates was obtained. Finally, the mean and the product of standard error of the mean were computed and the lower and upper confidence levels (LCL and UCL) for the comparison of each observed parameter were given by plotting the mean values of observed parameters from the original dataset against the decision lines. If any of the points plotted were outside the respective decision lines, hypothesis H_0,_ the means are homogenous, would be rejected at the α = 0.05, which would indicate that the means are not homogenous. A bootstrap t-test for a significant difference between two sample means was used to compare obtained data between control and LLLT group in each sampling point.

### Bioethical issues

The study protocol was approved by a local II Ethical Review Board in Wrocław (approval No. 9/2013). Each animal was provided with humane conditions and care according to the European Directive 2010/63/EU on the protection of animals used for scientific purposes.

## Results

All animals were successfully weaned from CPB and survived in good condition until the end of the study. There were no heart-lung machine hardware-related technical problems. Visual inspection ruled out thrombus formation in the bypass circuit.

**Figure 2:**
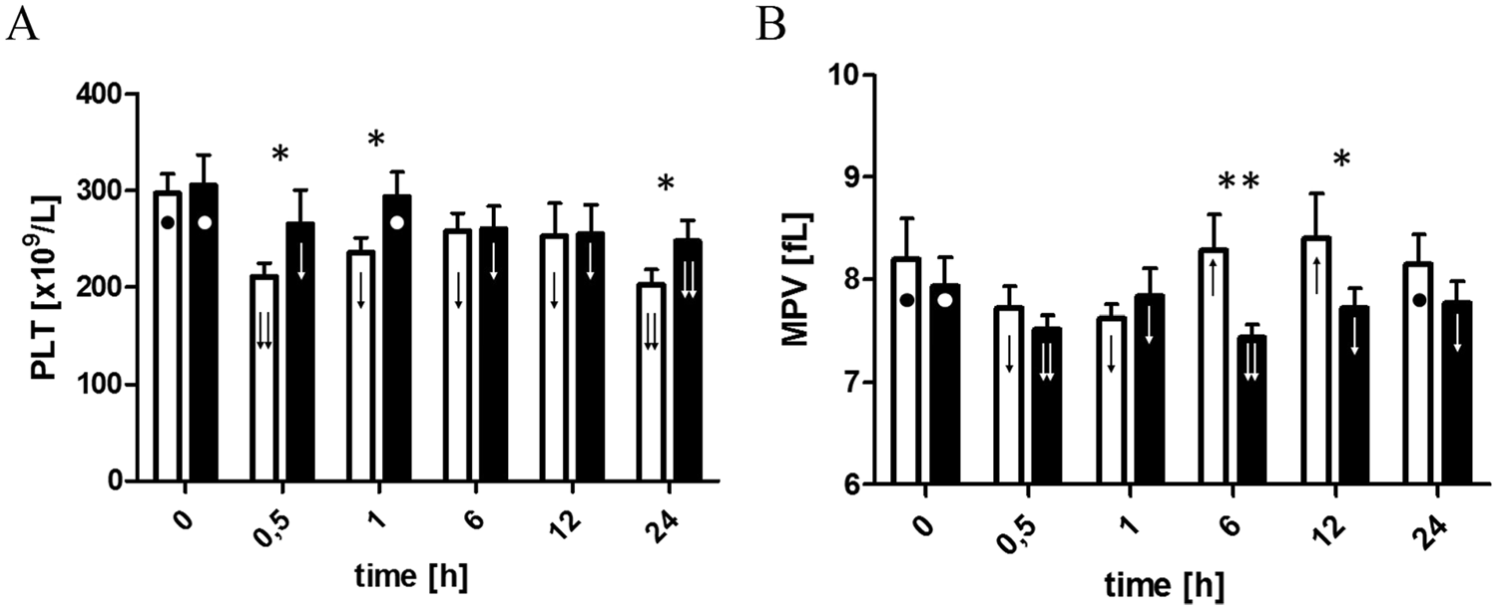
Changes in PLT count (**A**) and MPV (**B**) before initiation of ECC(T0), 30 minutes after initiation of ECC(T0.5), ACT and T1 in 6 (T6), 12 (T12), and 24 (T24) hours after initiation of ECC in the control (□) and LLLT group (■). Data are expressed as the mean ± SEM (n = 12/group). *p < 0.05, LLLT group compared with the control group at the sampling time. Changes in the values of the measurement points in a given group during the experiment were denoted by: ↓↓, ↓, ●, ↑. Values marked with the same symbol do not differ significantly.

**Figure 3:**
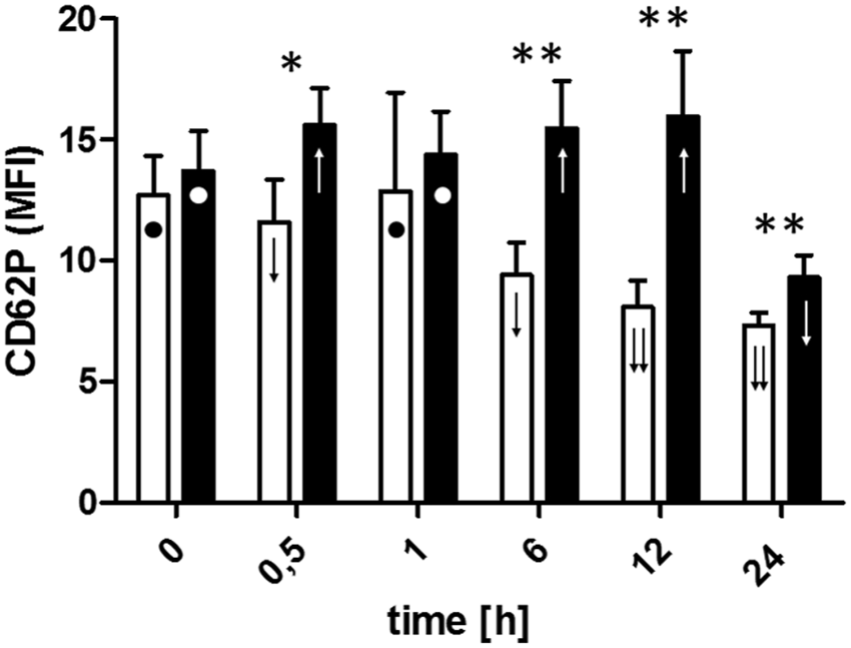
Changes in surface expression of PLT receptor CD62P before initiation of ECC(T0), 30 minutes after initiation of ECC(T0.5), post-circulation and normalization of ACT (T1) and 6 (T6), 12 (T12), and 24 (T24) hours after initiation of ECC in the control (□) and LLLT group (■). Data are expressed as the mean ± SEM (n = 12/group). *p < 0.05, **p < 0.01, LLLT group compared with the control group at the sampling time. Changes in the values of the measurement points in a given group during the experiment were denoted by: ↓↓, ↓, ●, ↑. Values marked with the same symbol do not differ significantly.

**Figure 4:**
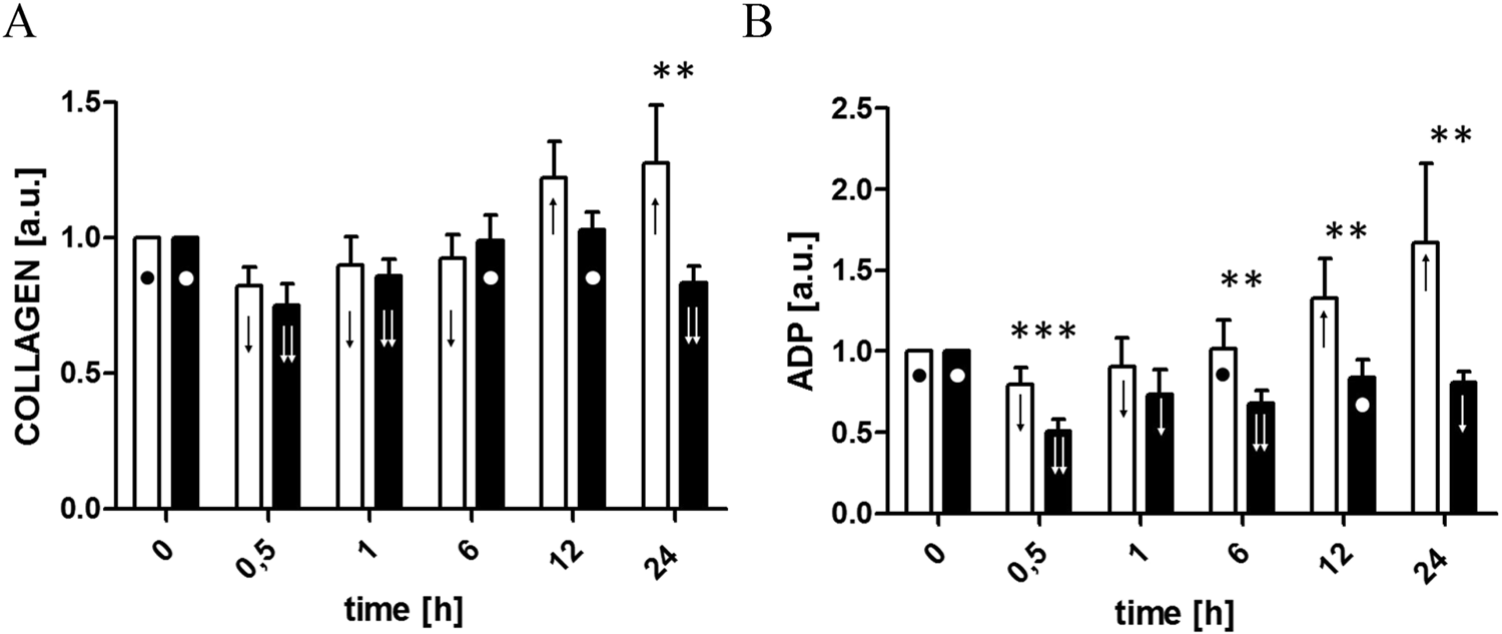
Changes in collagen-induced aggregation (**A**) and ADP-induced aggregation (**B**) with respect to the value obtained prior to commencement of ECC (T0), 30 minutes after initiation of ECC (T0.5), post-circulation and normalization of ACT (T1) and 6 (T6), 12 (T12), and 24 (T24) hours after initiation of ECC in the control (□) and LLLT group (■). Data are expressed as the mean ± SEM (n = 12/group). *p < 0.05, **p < 0.01, ***p < 0.001, LLLT group compared with the control group at the sampling time. Changes in the values of the measurement points in a given group during the experiment were denoted by: ↓↓, ↓, ●, ↑. Values marked with the same symbol do not differ significantly.

At the beginning of the experiment both groups were comparable in respect to the PLT number, their mean volume, expression of CD62P and aggregation induced by ADP and collagen. Changes in the above-mentioned parameters during the experiment are shown in the Figs 2–4.

### Changes in PLT count

In both groups, similar changes in PLT counts were observed during CPB. First, a significant decrease in PLT count after beginning CPB was noted (T0.5) and then the PLT number increased (T1). However, the absolute number of PLTs remained higher in the LLLT group, where the results were not different in T0 and T1. After weaning from CPB, the control group PLT counts remained constant until T12, and then finally demonstrated a significant drop by the end of the experiment (T24). In the LLLT group, the number of PLTs seen at the 6^th^ hour of the experiment was lower than after weaning from CPB but remained stable to the end of the experiment. Moreover, 24 hours after beginning CPB the number of PLTs was greater in the LLLT group than in controls (Fig. 2A).

### Changes in mean platelet volume (MPV)

During extracorporeal circulation, the MPV was reduced in both groups of animals. Increase in the MPV was seen in the first measurements after finishing CPB in the LLLT group and only at hour six of the experiment in the control group. Both in the 6^th^ and 12^th^ hour of the study, MPV was higher in the control group, so that after the observation period they were again comparable in both groups (Fig. 2B).

### Analysis of CD62P (P-selectin) expression

During the extracorporeal circulation, a slight decrease in CD62P expression was transiently observed, followed by a return to baseline after CPB in the control group. A significant reduction in CD62P expression was observed in further follow-up. The use of LLLT resulted in stabilization of CD62P expression for almost 12 hours of observation (when only results from T0.5 to T12 were taken into consideration, there was no statistical difference between CD62P expression between them – analysis not shown in the figure), but there was a significant decrease in expression at the 24 hour time point. In addition, from T1 to the end of the experiment, higher expression of CD62P was observed in the LLLT group compared to the control group (Fig. 3).

### PLT aggregation test

During CBP (T0.5) a significant reduction in PLT aggregation capacity in the presence of agonists (both collagen and ADP) was observed in both groups compared to baseline. In the control group, this trend lasted up to 6^th^ hour, followed by a rapid increase in aggregation capacity over the preoperative value. In the LLLT group, normalization of aggregation capacity was observed at 6^th^ hour when the agonist was collagen, and 12^th^ hour when ADP was used. Then, regardless of the agonist used, the aggregation decline was observed again at the 24^th^ hour time point (Fig. 4A,B).

## Discussion

In the present study, we aimed to evaluate whether blood irradiation with R/NIR light during CPB has beneficial effects on PLT function.

In our CPB model, we carefully selected the animal species. The physiology and anatomy of the porcine cardiovascular system and coagulation are similar to that of humans^20,21^. In addition, porcine PLTs share greater similarities in biochemical properties with human PLTs than other non-primate species^22,23^. Moreover, CPB is well-tolerated in pigs and the porcine model closely simulates the human situation in CPB.

The use of CPB is associated with risk for numerous complications related, inter alia, to physical damage or dysfunction of blood morphotic elements. The most important factor contributing to the hemostatic defect associated with CPB is generally considered to be an alteration in PLT function^24,25^. Extensive PLT loss may lead to hemostasis disorders and, consequently, to uncontrolled bleeding. On the other hand, in some patients, a reversal phenomenon of reactive thrombocytosis after cardiac operations is observed, which can predispose to myocardial infarction and vein graft occlusion in the early postoperative period^26^.

Human observational studies show that CPB induces a rapid decrease in PLT count within the first 5 min of circulation and that the largest deficit occurs within30 min of circulation^27,28^ and can last up to several days after surgery. In our study, we observed a similar relationship as PLT counts clearly decreased during CPB in both groups of animals, and the lowest number of PLTs was recorded after 30 minutes of ECC. A reduction in the number of PLTs during CPB may be the result of several mechanisms. First, blood contact with the artificial surface leads to PLT activation and changes in their shape, which promotes adhesion to the artificial perfusion circuit^29^. At the same time, activation of PLTs increase the likelihood of forming PLT-leucocyte aggregates. Secondly, lower PLT count in the circulating blood unit may be the result of a hemodilution with isotonic nonplasma fluids. Third possible mechanism - heparin-induced thrombocytopenia – seems unlikely as none of the animals had been exposed to heparin in the past and the time to produce antibody was relatively short.

We have shown that in the group of pigs which received the LLLT treatment, PLT count during ECC reached a higher value than in the control group^30^, (Fig. 1). Moreover, when only postoperative period was analyzed, PLT count in the LLLT group remained at a similar level, while in the control group droped at the end of the experiment. Considering that the hemodilution protocol and subsequent postoperative care of the animals were the same in both groups, differences were associated with the reduced destruction of PLT in the LLLT group during CBP.

In this study, we also monitored MPV. During the CPB we demonstrated a comparable decrease in MPV in both groups of pigs. Similar changes have been observed in studies conducted in humans treated with CPB^29,31^. As Guthikonda *et al*.^32^ and Mangalpally *et al*.^33^ showed, larger PLTs are more prone to activation and aggregation. Taking this into consideration, it seems that the initial decrease in MPV with a concurrent reduction in PLTs during ECC may be due, inter alia, to the loss of the larger and more reactive circulating blood PLTs, which adhere to the walls of the apparatus or may be damaged due to mechanical stress^31^. Differential MPV in the postoperative period may result from the constant exchange of the circulating PLT pool with new ones. The significant steady decline in MPV until the end of the CPB, with the subsequent significant MPV increase in the control group, may reflect initially significant PLT loss, followed by increased replacement by the young cells. In the LLLT group, these changes are less dynamic, and moreover, stabilization is visible after 12 hours. This may indicate, especially in comparison with relatively stable PLT counts, less PLT replacement (hence, less severe activation and PLT destruction).

We measured PLT activation by evaluation of activation-dependent surface receptor CD62P (also known as P-selectin) expression. In our experiment, CD62P expression in the LLLT group remained quite stable up to 12^th^ hour of observation, whereas in the control group we noted a significant and constant drop in CD62P expression after ECC till the end of observation. Reduced expression of P-selectin in the control group of pigs may be due to activation of PLTs and release of microparticles that carry CD62P receptors on their surface. Activated and degranulated PLTs rapidly lose surface P-selectin to the plasma pool, but continue to circulate^34^. This may explain the decrease in the expression of this molecule on PLTs in the control pigs. Moreover, activated PLTs expressing CD62P quickly form aggregates with circulating leukocytes, thus it is also possible that these receptors might not be detected^34^. The stable P-selectin level for the first 12 hours in the LLLT group indicates the stabilizing effect of R/NIR irradiation on the adhesion molecules on the surface of the PLTs.

Changes in PLT activation are related to their aggregation potential. Reilly *et al*.^35^ observed that in patients undergoing coronary artery surgery with the use of ECC, genes involved in PLT aggregation including GPIIb (ITGA2B), GPIIIa (ITGB3) and COX-1 (PTGS1), were up-regulated and overexpression was confirmed by flow cytometry analysis of the GPIIb/IIIa receptor. Moreover, Kobzar *et al*.^36^ showed that spontaneous aggregation was higher postoperatively than before surgery and increased through the observation period, being higher on the fifth day than on the third. We demonstrated a similar trend in the control group. Although we ceased the experiment after 24 hours, we noted elevated aggregation activity in the presence of agonists, both collagen and ADP, in the control group already at the 12^th^ hour of observation. In contrast, in the LLLT group, PLT aggregation was never higher than preoperatively, and the final result was even lower than the baseline one. On that basis, we can conclude that R/NIR irradiation reduced the ability of PLTs to form clots. Of course, one question that needs to be addressed is whether it has any clinical significance, and, if so, whether the phenomenon is beneficial, harmless or harmful. In cardiac surgery, especially in coronary artery bypass grafting (CABG), intense PLT aggregation may lead to serious sequelae related to thrombotic complications. As a standard of postoperative care after CABG, patients receive aspirin as an anti-PLT drug just for reduction of PLT activity. In that case, LLLT showed similar properties. On the other hand, diminished PLT function increases the risk of bleeding and inadequate post-injury clot formation. In our experiment, in both animal groups postoperative red blood cell parameters (hemoglobin concentration, hematocrit, red blood cell number at 24^th^ hour) were lower than baseline, but did not differ between groups^12^. This may suggest that different pattern of PLT activity in the LLLT group did not result in increased blood loss.

The evaluated results concerning PLT reaction to LLLT during and after CPB are consistent with the values that we obtained for other blood cells^12^. Most recently, for the same model as above, we showed that in the control group, a rapid systemic decrease in white blood cells (WBC) count during ECC was accompanied by a significant increase in red blood cells (RBC) membrane lipids peroxidation, while in the LLLT group the number of WBC and thiobarbituric acid reactive substances (TBARS) concentration both remained relatively constant, indicating limitation of the inflammatory process. And as a consequence, the hemolysis markers like plasma free hemoglobin, lactate dehydrogenase, and serum bilirubin concentration, were significantly reduced in the LLLT group. In this short-term experiment, neither significant differences in RBC count, and hemoglobin concentration nor adverse effects were detected. However, further trials with prolonged observation period are required to confirm that this new approach is safe and effective.

### The mechanism of LLLT impact on PLT function

The LLLT may decrease PLT activation during CPB directly, but also by the modification of the plasma proteins involved in blood-foreign surface interactions. Nevertheless, the exact molecular mechanism underlying this phenomenon is still controversial.

LLLT inhibits PLT activation by such agonists as ADP, collagen, epinephrine, ristocetin, PAF, fibrinogen, adrenaline, ristomycin, and TRAP^17,37–39^. As such, irradiation most likely disrupts the activation processes of the coagulation system at a level common to all receptors. Importantly, this process is dose-dependent and reversible^17,18^. There were identified two chromophores capable of absorbing the R/NIR radiation found in the PLTs: guanylate cyclase (GC) and nitric oxide synthase (NOs)^17^. Both contain hem, and NOs additionally has flavonic nucleotides as prosthetic groups^17^. In this case, activation of NOs results in NO formation and stimulation of GC, which, in turn, causes accumulation of cGMP in the cell. cGMP plays a role in regulating PLT activation, adhesion and aggregation and since R/NIR radiation has been shown to increase plasma cGMP concentrations, it can be concluded that this contributes to their inhibition.

Recently, Yang *et al*.^40^ demonstrated in the passive mouse immune thrombocytopenia model that exposure to LLLT significantly reduced PLT caspase-3 activation and the proportion of apoptotic PLTs to a near normal level as compared to the sham-light group. These results suggest that LLLT prevents PLTs from undergoing apoptosis and prolongs their lifespan in the presence of anti-PLT antibody. In this case, the mechanism underlying LLLT-mediated protection was associated with the ability of LLLT to preserve mitochondrial function, as the cytochrome c oxidase is thought to be one of the major photoacceptors of the R/NIR light^41^. Although the anti-PLT antibodies induce PLT apoptosis and result in mitochondrial membrane potential (ΔΨ_m_) depolarization^42^, LLLT treatment enhances mitochondrial function and maintains ATP production^43^. In our studies, we expected that blood contact with bioincompatible materials and non-physiological shear stress generated by perfusion circuit induced procoagulant PLT necrosis instead of apoptosis because it plays an important role in inducing inflammatory and repair processes, which are well known and routinely observed in CPB^44^. Nevertheless, we cannot exclude that the same mitochondrial mechanism was involved in PLT inhibition and preservation during CPB by R/NIR light.

What is also important, absorption of R/NIR radiation can significantly disturb the energy of hydrogen bonds, which, in turn, can lead to their disruption and the increased dissociation of water molecules^45,46^. The dehydration effect results in an alteration in the concentration of ions on the membrane surface. Charge modification results in changes in the entire structure of the cell membrane. R/NIR leads to conformational changes in membrane proteins or transport^45^, resulting in the activation of a cellular system, and the synthesis of the secondary messengers^47^. Such assumptions indicate that the cell membrane structure is involved in the modification of PLT activity by light.

## Conclusion

Limitation of platelet loss, pattern of aggregation and CD62P expression suggest that LLLT may stabilize platelet function during CPB and diminish the negative effects associated with the interaction of cells with an artificial surface. If the same effects were seen in human studies, LLLT could be a simple and safe method to diminish platelets destruction and destabilization during extracorporeal circulation, therefore limiting coagulation disorders and the need of platelet transfusion after cardiac surgery.

## Data Availability

The datasets generated during and/or analyzed during the current study are available from the corresponding author on reasonable request.
