# Publisher Correction: Low-level light therapy reduces platelet destruction during extracorporeal circulation

**DOI:** 10.1038/s41598-018-37391-z

**Published:** 2019-01-24

**Authors:** Anna Drohomirecka, Alicja Iwaszko, Tomasz Walski, Aleksandra Pliszczak-Król, Grzegorz Wąż, Stanisław Graczyk, Katarzyna Gałecka, Albert Czerski, Jolanta Bujok, Małgorzata Komorowska

**Affiliations:** 1 Department of Heart Failure and Transplantology, Institute of Cardiology, Warsaw, Poland; 2 Regional Specialist Hospital in Wrocław, Research and Development Centre, Wrocław, Poland; 3 Department of Immunology, Pathophysiology and Veterinary Prevention, Wrocław University of Environmental and Life Sciences, Wrocław, Poland; 4 Department of Biomedical Engineering, Wrocław University of Science and Technology, Wrocław, Poland; 5 “Medinet” Lower Silesian Centre for Heart Diseases, Wrocław, Poland; 6 Department of Animal Physiology and Biostructure, Faculty of Veterinary Medicine, Wrocław University of Environmental and Life Sciences, Wrocław, Poland

Correction to: *Scientific Reports* 10.1038/s41598-018-35311-9, published online 16 November 2018

The original version of this Article contained extensive errors in ordering of references in the Reference list.

In addition, this Article contained the following typographical errors in the reference citations.

In the Material and Methods section,

“Dose selection was based on the analysis of previously published data, including results of *in vitro* studies on the assessment of R/NIR radiation of different

spectral ranges, irradiance levels and exposure time of blood cells^6,7,8,9,10,11,12,13,14,15,16,17^.”

now reads:

“Dose selection was based on the analysis of previously published data, including results of *in vitro* studies on the assessment of R/NIR radiation of different spectral ranges, irradiance levels and exposure time of blood cells^6,7,8,9,10,11,12,13,14,15,16,17,18^.”

“Bootstrap analysis of variance was performed, as described previously, to evaluate whether changes in the values of individual parameters during ECC were significant^16,18^.”

now reads:

“Bootstrap analysis of variance was performed, as described previously, to evaluate whether changes in the values of individual parameters during ECC were significant^16,19^”

In the Discussion section,

“In our CPB model, we carefully selected the animal species. The physiology and anatomy of the porcine cardiovascular system and coagulation are similar to that of humans^19,20^. In addition, porcine PLTs share greater similarities in biochemical properties with human PLTs than other non-primate species^21,22^.”

now reads:

“In our CPB model, we carefully selected the animal species. The physiology and anatomy of the porcine cardiovascular system and coagulation are similar to that of humans^20,21^. In addition, porcine PLTs share greater similarities in biochemical properties with human PLTs than other non-primate species ^22,23^.

“The most important factor contributing to the hemostatic defect associated with CPB is generally considered to be an alteration in PLT function^23,24^. Extensive PLT loss may lead to hemostasis disorders and, consequently, to uncontrolled bleeding. On the other hand, in some patients, a reversal phenomenon of reactive thrombocytosis after cardiac operations is observed, which can predispose to myocardial infarction and vein graft occlusion in the early postoperative period^25^.”

now reads:

“The most important factor contributing to the hemostatic defect associated with CPB is generally considered to be an alteration in PLT function ^24,25^. Extensive PLT loss may lead to hemostasis disorders and, consequently, to uncontrolled bleeding. On the other hand, in some patients, a reversal phenomenon of reactive thrombocytosis after cardiac operations is observed, which can predispose to myocardial infarction and vein graft occlusion in the early postoperative period ^26^.”

“Human observational studies show that CPB induces a rapid decrease in PLT count within the first 5 min of circulation and that the largest deficit occurs within30 min of circulation^26,27^ and can last up to several days after surgery.”

now reads:

“Human observational studies show that CPB induces a rapid decrease in PLT count within the first 5 min of circulation and that the largest deficit occurs within30 min of circulation ^27,28^ and can last up to several days after surgery.”

“First, blood contact with the artificial surface leads to PLT activation and changes in their shape, which promotes adhesion to the artificial perfusion circuit^28^.”

now reads:

“First, blood contact with the artificial surface leads to PLT activation and changes in their shape, which promotes adhesion to the artificial perfusion circuit^29^.”

“We have shown that in the group of pigs which received the LLLT treatment, PLT count during ECC reached a higher value than in the control group^29^, (Fig. 1).”

now reads:

“We have shown that in the group of pigs which received the LLLT treatment, PLT count during ECC reached a higher value than in the control group ^30^, (Fig. 1).”

“As such, irradiation most likely disrupts the activation processes of the coagulation system at a level common to all receptors. Importantly, this process is dose-dependent and reversible^17,39^.”

now reads:

“As such, irradiation most likely disrupts the activation processes of the coagulation system at a level common to all receptors. Importantly, this process is dose-dependent and reversible^17,18^.”

“What is also important, absorption of R/NIR radiation can significantly disturb the energy of hydrogen bonds, which, in turn, can lead to their disruption and the increased dissociation of water molecules^43,46^.”

now reads:

“What is also important, absorption of R/NIR radiation can significantly disturb the energy of hydrogen bonds, which, in turn, can lead to their disruption and the increased dissociation of water molecules^45,46^.”

These errors have now been corrected in the HTML and PDF versions of the original Article.

